# Problems with analyses and interpretation of data in “use of the KDQOL-36™ for assessment of health-related quality of life among dialysis patients in the United States”

**DOI:** 10.1186/s12882-019-1609-2

**Published:** 2019-12-03

**Authors:** Ron D. Hays, John D. Peipert, Joel D. Kallich

**Affiliations:** 1 UCLA Department of Medicine, Division of General Internal Medicine & Health Services Research, 1100 Glendon Avenue, Suite 850, Los Angeles, CA 90024 USA; 2 Department of Medical Social Sciences, Feinberg School of Medicine, Northwestern University, 625 N. Michigan Ave, 27th floor, Chicago, IL 60611 USA; 3 Massachusetts College of Pharmacy and Health Sciences University, 179 Longwood Avenue, Boston, MA 02115 USA

**Keywords:** Renal disease, Health-related quality of life, KDQOL-36™

## Abstract

A recent article in the journal reported analyses of KDQOL-36™ survey data collected from 240,343 adults (330,412 surveys) dialyzed at a large dialysis organization in the United States during 2014–2016. The authors concluded that the KDQOL-36™ Symptoms and Problems of Kidney Disease scale had the highest mean score of the KDQOL-36™ scales. We note that this inference was erroneous because the scales are not scored on the same numeric scale. In addition, the authors found that responses to a general health perceptions item (“In general, would you say your health is excellent, very good, good, fair, or poor”) was not significantly associated with any of the 5 KDQOL-36 scale scores. In contrast, we find significant and noteworthy correlations in two other datasets. These analytic issues call into question the accuracy and validity of the conclusions of this paper.

Several recent studies have provided new and more extensive evidence of the psychometric soundness of the Kidney Disease Quality of Life (KDQOL™)-36 instrument including further support of its reliability and validity [1, 2] beyond what was reported in the original peer reviewed manuscript [3, 4]. In addition, the KDQOL has been shown to be predictive of healthcare utilization and mortality [5], outcomes that are important to patients who suffer from this end stage kidney disease (ESKD), their families, and health care providers who treat ESKD.

Cohen et al. analyzed KDQOL-36™ survey data collected from 240,343 adults (330,412 surveys) dialyzed at a large dialysis organization in the United States during 2014–2016. The authors reported the following mean scores on the 5 KDQOL-36 scales: Short-form (SF)-12 Physical Component Summary (PCS): 36.6; SF-12 Mental Component Summary (MCS): 49.0; Burden of Kidney Disease (BKD): 51.3; Symptoms and Problems of Kidney Disease (SPKD): 78.1; and Effects of Kidney Disease (EKD): 73.0. They concluded that the SPKD scale “had the highest mean score (78.1) of the 5 subscales on the KDQOL-36™” [6] (p. 4) and suggested that it exceeded the SF-12 PCS mean score by approximately 40 points. “Thus, the two scores convey very different messages about patient health: a PCS score in the 30’s is suggestive of extremely poor overall health, whereas an SPKD score of 70 or higher suggests a relatively low symptom burden. This pattern is suggestive, although not proof positive, that the SPKD subscale may be topped out” [6] (p. 7).

Cohen et al. [6] do not account for the fact that the SF-12 PCS and MCS are not on the same numeric scale as the BKD, SPKD, and EKD. The SF-12 PCS and MCS are scored on a T-score metric, which has a mean of 50 and standard deviation of 10 in the U.S. general population, while the kidney-targeted scales are scored on a 0–100 possible range and have variable means and standard deviations. Therefore, scores on the SF-12 scales and the kidney-targeted scales are not directly comparable to justify their conclusion. A PCS score of 36.6 does indicate a physical health related quality of life score nearly a standard deviation and a half below the US general population, but it cannot be directly compared with the SPKD scale that has a 0–100 possible range.

In fact, each of the means reported by Cohen et al. [6] are very similar to those recently reported for the KDQOL-36 United States dialysis population [7], as shown in Table 1 below. To aid in score interpretation, users of KDQOL instruments are encouraged to refer to [7] and the publicly available and free scoring guide: https://www.rand.org/health-care/surveys_tools/kdqol.html.

**Table 1:** Mean KDQOL-36 Scale Scores in Cohen et al. [6] and Peipert et al. [7]

KDQOL-36 Scale	Cohen et al. [6]	Peipert et al. [7]
SF-12 Physical Component Summary	36.6	37.8
SF-12 Mental Component Summary	49.0	50.9
Burden of kidney disease	51.3	52.8
Symptom/problems	78.1	79.0
Effects of kidney disease	73.0	74.1

Consistent use of norm-based scoring in the future is preferred because it would help make the direct comparisons desired in Cohen et al.’s [6] paper and other, similar applications. It facilitates comparisons because the referent norm (e.g., general population) is built into the scoring algorithm. For example, T-scores above 50 are better than, and those below 50 are worse than, a referent population for measures scored in a positive direction (higher score is better). Furthermore, since the standard deviation for each scale is standardized to be at 10, it is easy to see exactly how far above or below a score is from the norm in standard deviation units.

One of the questions in the KDQOL-36 is the often-used general health rating item [8]. Cohen et al. [6] stated that the response to that item (“In general, would you say your health is”) “was not correlated with any of the 5 subscale scores” [6] (p. 7). These results are improbable and inconsistent with prior research. For example, correlations based on the Medical Education Institute dataset [7] and another dataset (Peipert JD, Caicedo JC, Friedewald JJ, et al: Trends and predictors of multidimensional health-related quality of life after living donor kidney transplantation, submitted) that included 506 patients who completed the KDQOL-36 at the time of evaluation for transplant (before transplant surgery) show highly significant and noteworthy associations. Table 2 shows product-moment correlations (Spearman rank-order correlations were similar).

**Table 2:** Product-moment correlations of the general health item with the KDQOL-36 scales

	In general, how would you rate your health? (Medical Education Institute)	In general, how would you rate your health? (Transplant data)
SF-12 Physical Component Summary	0.56	0.53
SF-12 Mental Component Summary	0.34	0.29
Burden of Kidney Disease	0.35	0.39
Symptom and Problems of Kidney Disease	0.37	0.34
Effects of Kidney Disease	0.34	0.32

It is possible that there was an error in Cohen et al.’s [6] scoring or analysis of the KDQOL-36. However, these suspect correlations and the misinterpretation of KDQOL-36 scale scores noted above call into question the validity of all the analyses and conclusions reached in this manuscript. While errors such as these are sometimes made by researchers unfamiliar with the instrument being employed, the field relies on peer review to discover these flaws prior to publication.

Finally, Cohen et al. suggested that “new or revised HRQOL assessment tools may be designed to addressed those factors that are most important to dialysis patients” and that “improved instruments may in turn provide a more robust foundation to guide interventions aimed at improving HRQOL in patients with ESRD” [6] (p. 8). Previous published articles provide concrete ways to improve the KDQOL-36. For example, Peipert and colleagues [9–11] suggested replacing the SF-12 PCS and MCS with the Patient-Reported Outcomes Measurement Information System (PROMIS®) measures [12–14].

We agree that improvements to patient-reported outcome measures like the KDQOL-36 are worthwhile. These efforts often occur iteratively, across multiple analyses of diverse datasets like the one analyzed by Cohen, et al. [6]. However, recommendations for improvement to the KDQOL-36 need to be based on accurate and appropriate statistical analyses and interpretation of the scores. The KDQOL Working Group (http://www.kidney.org/sites/default/files/docs/cnsw_webinar_kdqol-36_final_1.pdf) is available to analyze datasets and work with those with access to datasets to ensure the accuracy of results and validity of the interpretation of the scale scores. A third-party analysis of KDQOL data held by private organizations provides a counterbalance to the perceived or actual financial conflict of interest and lack of transparency: “The datasets generated and/or analyzed during the current study are not publicly available due fact (sic) that they are derived from the proprietary database of a large dialysis organization” [6] (p. 8). Working with researchers from academia with high levels of training and experience in patient-reported outcomes research and psychometrics should be embraced to both raise the quality of all research work and assist in the training of future professionals in the field. Such collaborations between industry and academia are often fruitful and stand to make a significant, positive impact on ESKD patients’ lives.

## Data Availability

Requests for the datasets analyzed in the current study can be made to the authors except by those who restrict access to their data.

## Abbreviations

BKD: Burden of kidney disease
KDQOL™: Kidney Disease Quality of Life
MCS: Mental Component Summary
PCS: Physical Component Summary
PROMIS®: Patient-Reported Outcomes Measurement Information System
SPKD: Symptoms/problems of kidney disease
